# Analysis of clinical characteristics of breast cancer patients with the Japanese founder mutation *BRCA1* L63X

**DOI:** 10.18632/oncotarget.26852

**Published:** 2019-05-14

**Authors:** Reiko Yoshida, Chie Watanabe, Shiro Yokoyama, Mayuko Inuzuka, Junko Yotsumoto, Masami Arai, Seigo Nakamura, the Registration Committee of the Japanese HBOC consortium

**Affiliations:** ^1^ Division of Clinical Genetic Oncology, Cancer Institute Hospital, Tokyo, Japan; ^2^ Division of Breast Surgical Oncology, Department of Surgery, Showa University School of Medicine, Tokyo, Japan; ^3^ Department of Nursing, Faculty of Human Sciences, Sophia University, Tokyo, Japan; ^4^ The 3rd Department of Breast Cancer, Tianjin Medical University Cancer Institute and Hospital, National Clinical Research Center for Cancer, Tianjin, China

**Keywords:** hereditary breast and ovarian cancer, BRCA1, founder mutation, L63X, c.188T>A

## Abstract

**Background:** *BRCA1* and *BRCA2* are high-penetrance inherited genes; different founder mutations have been reported in various areas and races. By using trial registration data from the Japanese hereditary breast and ovarian cancer syndrome (HBOC) consortium, we aimed to explore the clinicopathological characteristics of breast cancer patients with the Japanese founder mutation *BRCA1* L63X.

**Results:** We found 88 *BRCA1* carriers, 76 *BRCA2* carriers, and one carrier of both *BRCA1* and *BRCA2*. Of 46 independent *BRCA1* mutations, the *BRCA1* L63X mutation was detected in 26 patients. We observed a significant difference in the proportion of triple-negative breast cancer phenotype among 88.9%, 72.5%, and 26.8% of *BRCA1* L63X mutation, *BRCA1* mutation, and *BRCA2* mutation carriers, respectively (*p* < .001). Additionally, significant differences were also observed in nuclear grade in the resultant breast cancer between the groups (*p* < .001).

**Conclusions:** A high proportion of Japanese HBOC patients showed the *BRCA1* L63X mutation, and the clinical characteristics of breast cancer in patients with this mutation might differ from those in patients with other *BRCA1* or *BRCA2* mutations, in terms of the subtype and nuclear grade of the resultant cancer.

**Methods:** From 827 patients in the Japanese HBOC consortium through August 2015, patients with *BRCA1/2* mutations were included in this study. We compared the clinicopathological features among patients with *BRCA1* L63X mutation, other *BRCA1* mutations, and *BRCA2* mutations using Chi-square test.

## INTRODUCTION

The number of breast cancer patients is increasing every year, and the incidence of breast cancer is the highest (1 in 11 [9%]) among Japanese women according to the Center for Cancer Control and Information Services of the National Cancer Center [1]. In general, approximately 5%–10% of all breast cancers develop because of a genetic predisposition [2, 3].

Individuals with hereditary breast and ovarian cancer syndrome (HBOC), which is caused by germline pathogenic variants of *BRCA1* or *BRCA2*, have an increased risk for breast cancer, ovarian cancer, prostate cancer, and pancreatic cancer [4–8]. In hereditary cancer, racial differences are important. For example, in persons of Ashkenazi Jewish heritage, three germline variants are observed: c.68_69delAG (*BRCA1*), c.5266dupC (*BRCA1*), and c.5946delT (*BRCA2*) [9]. As many as one in 40 Ashkenazi Jews have one of these three variants. A founder mutation is defined by the National Cancer Institute as “a gene mutation observed with high frequency in a group that is or was geographically or culturally isolated, in which one or more of the ancestors was a carrier of the mutant gene” [10]. Some reports on founder mutations of HBOC have been published from studies performed in Asia. From the Korean Hereditary Breast Cancer study [11] performed in Korea, one *BRCA2* pathogenic variant (c.7480C>T) was identified as the Korean founder mutation using data from over 3,000 breast cancer patients [12]. Furthermore, using data from 651 breast cancer patients in Southern China, one *BRCA1* pathogenic variant (c.981_982delAT) and three *BRCA2* pathogenic variants (c.3109C>T, c.7436_7805del370, and c.9097_9098insA) were analyzed and reported as Chinese founder mutations [13]. In Japan, only a few reports on founder mutations of HBOC have been published [14–16]. The c.188T>A (307T>A) variant, which causes the amino acid change L63X, was reported as the Japanese founder mutation in breast cancer and ovarian cancer. *BRCA1* L63X mutation was detected in Eastern Japan according to a previous report [15]. However, the data of these studies were not obtained from large study populations.

Recently, the Japanese HBOC consortium was established [16]. In this study, we aimed to analyze and report the clinical characteristics of breast cancer patients with *BRCA1* L63X mutation, which is one of the *BRCA1* founder mutations in the Japanese population, using the Japanese HBOC consortium trial registration data.

## RESULTS

**Figure 1:**
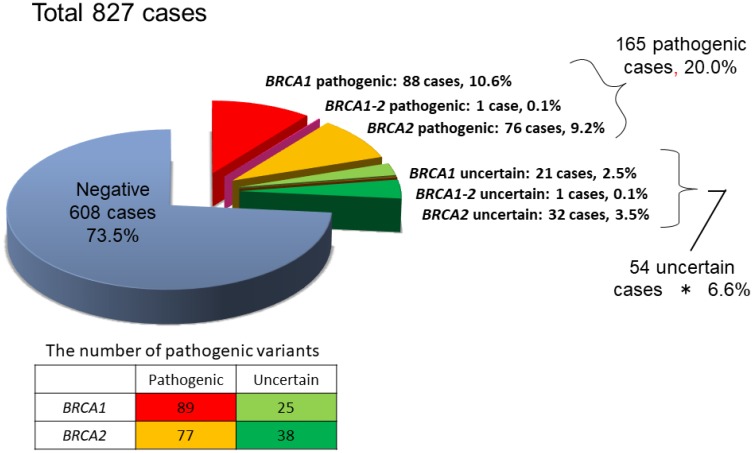
Results of *BRCA1/2* genetic testing of 827 patients.

We collected the data of 827 patients (752 breast and/or ovarian cancer patients and 75 patients without these cancers) who had undergone genetic testing in this trial registration through August 2015. The data showed 88 *BRCA1* carriers, 76 *BRCA2* carriers, one carrier of both *BRCA1* and *BRCA2*, 54 variants of uncertain significance, and 608 non-carriers. We detected 46 independent pathogenic variants (including pathogenic and probably pathogenic variants) of *BRCA1* and 52 independent pathogenic variants (including pathogenic and probably pathogenic variants) of *BRCA2* (Figure 1). The *BRCA1* pathogenic variants identified in three or more patients were c.188T>A (L63X), c.2800C>T, c.2389delGA, and c.3441delG (Table 1). The *BRCA1* L63X mutation was detected in 26 patients (30.6% of *BRCA1* carriers and 16.3% of *BRCA1/2* carriers). The *BRCA2* pathogenic variants identified in three or more patients were c.5576_5579delTAA, c.1278delA, c.8504C>A, c.9117G>A, c.6952C>T, and c.8589insA (Table 2).

**Table 1:** *BRCA1* pathogenic variants (identified in ≥three or more patients)

Exon	AA change	BIC nomenclature	HGVS nomenclature	Effect on AA	Mutation pattern	Number
5	L63X	307T>A	c.188T>A	p.Leu63Ter	nonsense	26
11C	Q934X	2919C>T	c.2800C>T	p.Glu934Ter	nonsense	6
11B	STOP799	2508delGA	c.2389delGA	p.Glu797Thrfs^*^3	deletion	4
11D	STOP1154	3561delG	c.3442delG	p.Glu1148Argfs^*^7	deletion	4

Abbreviations: BIC: breast cancer information core, HGVS: human genome variation society, AA: amino acid.

**Table 2:** *BRCA2* pathogenic variants (identified in ≥three or more patients)

Exon	AA change	BIC nomenclature	HGVS nomenclature	Effect on AA	Mutation pattern	Number
11E	STOP1862	5804del4	c.5576_5579delTTAA	p.lle1859Lysfs	Deletion	8
10	STOP429	1506delA	c.1278delA	p.Asn433Glnfs	Deletion	5
20	S2835X	8732C>A	c.8504C>A	p.Ser2835Ter	Nonsense	4
23	P3039P	9345G>A	c.9117G>A	p.Pro3039Pro	Silent	4
12	R2318X	7180C>T	c.6952C>T	p.Arg2318Ter	Nonsense	3
20	STOP2868	8817insA	c.8589dupA	p.Ala2864Serfs^*^5	Insertion	3

To clarify the *BRCA1* L63X founder mutation, we compared the three groups (*BRCA1* L63X, other *BRCA1* mutations, and *BRCA2* mutations) of breast cancer patients (Table 3). Comparison between the development and type of breast cancer and ovarian cancer in the probands showed that patients with both breast and ovarian cancers were observed only in the other *BRCA1* mutation-positive group, although no significant difference was observed. The age at breast cancer onset was 40.3 years, 40.4 years, and 41.6 years in the *BRCA1* L63X mutation-positive group, other *BRCA1* mutation-positive group, and *BRCA2* mutation-positive group, respectively. In general, although patients with *BRCA1* mutation-positive breast cancer were younger than those with *BRCA2* mutation-positive breast cancer [4], there was no significant difference in the age of onset and the status of breast cancer between the three groups. In addition, with regard to the breast cancer type, multifocal breast cancer was observed only in the *BRCA2* mutation group, and there was no significant difference among the three groups. The results of the comparison of the breast cancer subtypes between the three groups were as follows: triple-negative breast cancer accounted for 89% of the *BRCA1* L63X mutation group, 64.4% of other *BRCA1* group, and 21.2% of *BRCA2* group (*p* < 0.001). Significant differences in nuclear grade was also observed among the three groups (*p* < 0.001). During genetic counseling, we asked the patients about a family cancer history up to at least second-degree relatives. In the West, it was reported that the ovarian cancer risk was higher in the *BRCA1* mutation group than in the *BRCA2* mutation group, while the prostate cancer risk was higher in the *BRCA2* group than in the *BRCA1* group. However, no significant difference was observed between groups in terms of family cancer histories.

**Table 3:** Characteristics of three groups of breast cancer patients

	*BRCA1* L63X *n* = 25^a^	Other *BRCA1* mutation *n* = 59	*BRCA2* mutation *n* = 76^a^	*p*-value	*BRCA1/2* negativec^c^ *n* = 590
Cancer development					
BC only (%)	22 (88)	53 (89.8)	73 (96.1)		577 (97.8)
OC only (%)	3 (12)	2 (3.4)	3 (3.9)		8 (1.4)
BC+OC (%)	0	4 (6.8)	0		5 (0.8)
				NS	
BC onset					
Age	40.3	40.4	41.6		45.2
(range)	(28–74)	(23–71)	(19–71)		(22–85)
				NS	
BC situation^b^					
Single BC (%)	21 (95.5)	55 (96.5)	65 (90.3)		468 (81.0)
Bilateral BC (%)	1 (4.5)	2 (3.5)	3 (4.2)		87 (15.1)
Multiple BC (%)	0	0	4 (5.6)		23 (3.9)
				NS	
BC subtype^b^					
Luminal (%)	2 (11)	9 (20)	23 (44.2)		311 (62.4)
Luminal+HER2 (%)	0	2 (4.4)	6 (11.5)		42 (8.4)
HER2 (%)	0	0	1 (1.9)		28 (5.6)
Triple negative (%)	16 (89)	29 (64.4)	11 (21.2)		117 (23.5)
				*p =* < 0.001	
BC nuclear grade^b^					
1	0	3 (8.3)	21 (37.5)		227 (59.3)
2	5 (31.2)	5 (13.9)	18 (32.1)		121 (31.6)
3	11 (68.8)	28 (77.8)	17 (30.4)		135 (35.2)
				*p =* < 0.001	
Family history					
BC (average number)	2.31	2	2.75		0.97
(range)	(0–5)	(1-6)	(0-6)		(0-5)
OC (average number)	0.54	0.81	0.36		0.15
(range)	(0-3)	(0-4)	(0-3)		(0-3)
PC (average number)	0.154	0.145	0.2		0.1
(range)	(0–1)	(0–2)	(0–2)		(0–2)
				NS	

^a^Excluding one carrier of both the *BRCA1* (L63X) and *BRCA2* mutations.

^b^Excluding unknown data.

^c^Including VUSs (Variants of Uncertain) and benign variants.

Abbreviations: BC: breast cancer, OC: ovarian cancer, PC: prostate cancer.

## DISCUSSION

*BRCA1* was discovered in 1994 and *BRCA2* in 1995 [18, 19]. In the United States, *BRCA1/2* clinical testing started in 1996. However, this clinical testing started in 2006 in Japan, although testing had been performed in research studies. Compared to Western countries, because an HBOC medical treatment system started later in Japan, only a few reports on HBOC from Japan were obtained. Sugano *et al*. [20] reported that 36 of the 135 proband patients were *BRCA1/2* mutation carriers including five *BRCA1* L63X mutation carriers. It also described the prevalence and clinical characteristics of HBOC in Japan in 2008. Nakamura *et al*. [21] reported that 80 of the 260 proband patients were *BRCA1/2* mutation carriers including 10 *BRCA1* L63X mutation carriers and described the prevalence and differentiation of HBOC. Our study included the largest number of HBOC patients reported in Japan. The *BRCA1* L63X mutation, which was reported only in Asians in the Breast Cancer Information Core database [22], was identified as a Japanese founder mutation of ovarian cancer in haplotype analysis. In our study, we used multi-facility trial registration data; thus, we have not performed haplotype analysis, but it is suggested that it is a common ancestor. Considering that it is the most frequent HBOC founder mutation among Japanese people, previous analytical studies did not have large sample sizes (Table 4). In this study, we aimed to confirm that a high proportion of Japanese HBOC patients showed the *BRCA1* L63X mutation.

**Table 4:** Comparison of reports on the *BRCA1* L63X mutation

	Institution	Number of *BRCA* genetic testing	Number of *BRCA1* mutations	Number of *BRCA1* L63X	Analysis	Results
Ikeda 1997 [14]	Osaka Univ.	113	15	4	Haplotype analysis	Common ancestors
Sekine 2001 [15]	Niigata Univ.	82	24	7	Clinicopathological Analysis	Lower proportion of advanced
Nagata 2001 [17]	Niigata Univ.	45 (*BRCA1* only)	24	7	Haplotype analysis (Data not shown)	Common ancestors
Momozawa 2018 [34]	Biobank Japan	BC case: 7,051 Control: 11,241	102	26	Allele frequency	26% in *BRCA1* mutations
Our data	HBOC consortium	827	89	25	Clinicopathological analysis	Higher of TN proportion

Considering that hereditary cancer differs according to ethnicity, a founder mutation is an important element and leads to questions such as “How many founder mutations are observed in a related gene?” and “What kinds of effects does the genotype of a founder mutation have on cancer phenotype?” Identification of a founder mutation and knowledge of its prevalence in each population provides important information for genetic counseling, cancer risk assessment, and the development of a cost-effective screening strategy. Many hypotheses concerning Japanese ancestry have been proposed. Recently, using the Y chromosome, which does not undergo genetic recombination, haplotype research, and a genome-wide single nucleotide polymorphism database, the Japanese were believed to be a mixed population that descended from the Jomon people (hunters), who originated in Southeast Asia 10,000 years ago and settled in the Japanese archipelago, and the Yayoi people (farmers), who emigrated from the East Asian continent 2,000 to 3,000 years ago [22–26]. The Japanese population is the largest in the world to have inherited the haplotype of the Jomon people, which is not seen in Chinese and Korean populations [27]. This might be because Japan could be considered as an island country at the outskirts of the eastern countries. As a result, we could consider that the frequency of the *BRCA1* L63X mutation might be very high in the Japanese population (Table 5). However, the Japanese population might be mixed: one c.7480C>T *BRCA2* variant (the Korean founder mutation) and one c.470-471delCT *BRCA1* variant (the Southern Chinese founder mutation) were observed in this registry [16].

**Table 5:** Proportions of founder mutations according to race

Race	Founder mutation	*BRCA1/2* carriers	Proportion of founder mutation (%)
Korea [12]	*BRCA2 c. 7480C>T*	148	18 (12.2)
Southern Chinese [13]	*BRCA1 c.981_982delAT*	69	2 (2.9)
	*BRCA2 c.3109C>T*		10 (14.5)
	*BRCA2 c.7436_7805del370*		2 (2.9)
	*BRCA2 c.9097_9098insA*		2 (2.9)
Mainland Chinese [28]	*BRCA1 c.981_982delAT*	566	18 (3.2)
	*BRCA2 c.3195_3198delTAAT*		5 (0.9)
	*BRCA2 c.5576_5579delTTAA*		5 (0.9)
Japanese (our study)	*BRCA1 c.188 T>A*	161	26 (16.1)

In general, the population-adjusted age distribution of breast cancer patients in Japan showed two distinct peaks, one for patients in their late 40s and another for patients in their early 60s [29]. However, according to the Surveillance, Epidemiology, and End Results database [30], between 2009 and 2013, in the age distribution of newly diagnosed breast cancer patients in the USA, there was only one peak for patients in their early 60s; in contrast, there was one peak for patients in their 40s in this age distribution in South Korea [31]. These findings indicated that there are two mixed peaks in Japan: one in patients in their 40s, similar to East Asian women, and another in patients in their early 60s, similar to Western women. In our result, the average age at breast cancer onset between the *BRCA1* group and *BRCA2* group was nearly equal.

According to the Consortium of Investigators of Modifiers of *BRCA1/2*, regarding the pathology of breast cancer with *BRCA1/2* mutations [32], triple-negative breast cancer accounted for 69% of *BRCA1* mutations and 16% of *BRCA2* mutations, and the nuclear grades (grade 1, grade 2, and grade 3 [%]) were 3%, 20%, and 77% for *BRCA1* mutations and 7%, 43%, and 50% for *BRCA2* mutations, respectively. Although the pathological features of *BRCA1* breast cancer and *BRCA2* breast cancer reported in our study were similar to that of Western studies, the rate of triple-negative breast cancer was very high (89%) in the *BRCA1* L63X mutation group. The proportion of patients with triple negative breast cancer among those who were *BRCA1/2* negative in this study was 28.9% (173/597), which is higher than that of 15.4% found in the registered data of the Japan Breast Cancer Society [33]. The triple negative subtype appeared to be a part of the *BRCA* genetic testing criteria; however, the proportion of patients with triple negative breast cancer in those with an L63X and *BRCA1* positive status in our study was much higher than that in those who were *BRCA 1/2* negative. This trend was also seen in research published overseas. Because of modifiers (including genetic background and environmental factors), the characteristics of breast cancer harboring a *BRCA1/2* mutation in the Japanese population might be different from that of other races. Hence, we need to create a Japanese HBOC database and elucidate the realities of HBOC breast cancer in Japan.

The association between the type and location of *BRCA1* and *BRCA2* mutations was published in 2015 by Rebbeck *et al*. [34], and they identified that breast cancer cluster regions (BCCR) were locations where carriers had a tendency to develop breast cancer mutations. The *BRCA1* L63X mutation is located in the putative BCCR1 region, which has been reported in the juvenile-onset region in this study [34]. However, in this study, data obtained from the Asian population entailed that only 0.2% of individuals were *BRCA1* carriers.

Recently, germline pathogenic variants of 11 breast cancer genes were explored in Japanese breast cancer patients using the samples from Biobank Japan, which is a multi-institutional hospital-based registry that collects DNA from peripheral blood leukocytes of patients with various common diseases, including breast cancer regardless of the possibility for HBOC [35]. They also found recurrent *BRCA1* L63X founder mutation in *BRCA1* pathogenic variants (26/102: 26% OR = 20.8) in the unselected breast cancer patients. This tendency might be found not only in East Japan but also in Japan as a whole.

In this trial registration, some selection bias might have occurred. Because the registration committee members of three hospitals belonged to the breast cancer division, we might have had a tendency to register more breast cancer patients than ovarian cancer patients. In this registration, although most patients matched the criteria for genetic testing according to the National Comprehensive Cancer Network guidelines [36], no standards were indicated in the registry. This might have affected the comparison studies including those on the age of onset, development and type of breast and ovarian cancer, clinical characteristics of breast cancer, and family cancer history.

In conclusion, the founder mutation *BRCA1* L63X was observed in approximately 30% of *BRCA1* carriers and is frequently observed among Japanese patients. This could be attributed to the fact that Japan is an island. The clinical characteristics of breast cancer in patients with the *BRCA1* L63X mutation might not differ from those caused by other *BRCA1* or *BRCA2* mutations, except the subtype and nuclear grade of the resultant cancer. Further investigation is required to appropriately validate and obtain the data. Hence, we started a nationwide registration in March 2016 [37].

## MATERIALS AND METHODS

**Figure 2:**
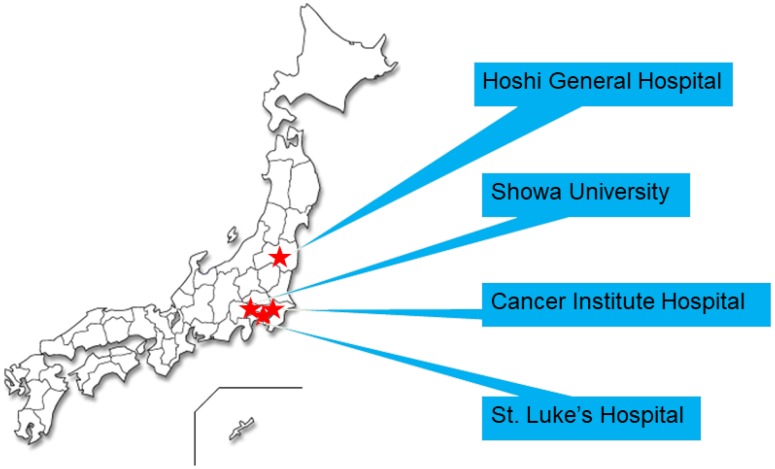
Trial registration committee members of four hospitals. As shown in the map, the four hospitals are located in Eastern Japan.

This investigation has been conducted in accordance with the ethical standards, the Declaration of Helsinki, and national and international guidelines. This study was approved by the Ethical Review Boards of the Japanese HBOC Consortium and each medical institution. Informed consent has been obtained in principle [16]. The Japanese HBOC consortium was established in 2012, following another study conducted by the Japanese Breast Cancer Society (2010–2012). This consortium aimed to raise awareness of HBOC in Japan and to provide an effective healthcare system for HBOC patients and their families. Their activities included constructing the Japanese HBOC database, promoting educational activities about HBOC, and developing HBOC guidelines and research. At first, for constructing the Japanese HBOC database, a trial registration was conducted by the registration committee members of four hospitals, namely, Hoshi General Hospital (Fukushima), Showa University Hospital (Tokyo), Cancer Institute Hospital (Tokyo), and St. Luke’s International Hospital (Tokyo) (Figure 2). We registered patients who underwent genetic testing at these four hospitals [16].

During the registration, the following data were collected: family identification number, birthdate, sex, age at death, development and type of breast cancer and ovarian cancer, history of other cancers, age during first childbirth, age at menarche, age at menopause, cause of death, data about breast cancer (age at onset, location, discovery opportunities, treatments, operative method, operation date, pathological histology, tumor size, nuclear grade, estrogen receptor status, progesterone receptor status, human epidermal growth factor receptor 2 status, and Ki-67 index score), data about ovarian cancer (age at onset, date of treatment initiation, treatment result, location, treatments, operative method, operation date, pathological histology, stage, and nuclear grade), date of genetic testing, result of genetic testing, genotype, prophylactic surgery, and family history (at least till second-degree relatives). The registration data were updated annually. All genetic tests were performed by FALCO HOLDINGS, which signed an exclusive contract with Myriad Genetics (United States) and holds the *BRCA1/2* genetic testing patent in Japan. Genetic testing for *BRCA1/2* was performed via direct sequencing and multiplex ligation-dependent probe amplification.

From all the registered patients, we evaluated the age at breast cancer onset, pathological features, clinical features, and family history of breast cancer patients, and we compared the difference between those with the *BRCA1* L63X mutation, other *BRCA1* mutations, and *BRCA2* mutations using the Chi-square test.

## Abbreviations

HBOC: hereditary breast and ovarian cancer syndrome
BCCR: breast cancer cluster regions
